# Supplementary material for: Comparative genomics provides new insights into the diversity, physiology, and sexuality of the only industrially exploited tremellomycete: Phaffia rhodozyma
Source: BMC Genomics. 2016 Nov 9;17:901. doi: 10.1186/s12864-016-3244-7 (PMC5103461; doi:10.1186/s12864-016-3244-7)
Supplement: Additional file 6: — List of orphan genes with links to PFAM (related to Additional file 1: Table S1). (ZIP 1428 kb) [file 12864_2016_3244_MOESM6_ESM.zip › PFAM_UNIQUE/G04010_P.html]

#                                                                            --- full sequence --- -------------- this domain -------------   hmm coord   ali coord   env coord
  
# target name        accession   tlen query name           accession   qlen   E-value  score  bias   #  of  c-Evalue  i-Evalue  score  bias  from    to  from    to  from    to  acc description of target
  
#------------------- ---------- ----- -------------------- ---------- ----- --------- ------ ----- --- --- --------- --------- ------ ----- ----- ----- ----- ----- ----- ----- ---- ---------------------
  
G04010\_P             -            256 DUF1421              PF07223.6    358       3.8    5.9   9.6   1   1     0.019       4.9    5.5   9.6   116   230    57   170     9   185 0.72 - Protein of unknown function (DUF1421)  
G04010\_P             -            256 DUF605               PF04652.11   380       6.4    4.7  10.0   1   1      0.12       7.3    4.5  10.0   193   325    16   158     4   231 0.63 - Vta1 like  
G04010\_P             -            256 Fork\_head\_N          PF08430.7    136       6.7    5.8   7.9   1   1    0.0073        11    5.1   7.7    38    98    97   155    54   181 0.69 - Forkhead N-terminal region  
G04010\_P             -            256 Mating\_C             PF12737.2    419      0.83    7.1   8.6   1   1    0.0028       1.2    6.6   8.6   167   252    77   165    61   184 0.77 - C-terminal domain of homeodomain 1
